# Polymer Optical Fiber-Based Integrated Instrumentation in a Robot-Assisted Rehabilitation Smart Environment: A Proof of Concept

**DOI:** 10.3390/s20113199

**Published:** 2020-06-04

**Authors:** Arnaldo Leal-Junior, Leticia Avellar, Jonathan Jaimes, Camilo Díaz, Wilian dos Santos, Adriano A. G. Siqueira, Maria José Pontes, Carlos Marques, Anselmo Frizera

**Affiliations:** 1 Graduate Program of Electrical Engineering, Federal University of Espirito Santo, Vitória 29075-910, Brazil; leticia.avellar@aluno.ufes.br (L.A.); c.rodriguez.2016@ieee.org (C.D.); mjpontes@ele.ufes.br (M.J.P.); frizera@ieee.org (A.F.); 2 Department of Mechanical Engineering, Engineering School of São Carlos, University of São Paulo, São Carlos 13566-590, Brazil; jonathancj@usp.br (J.J.); wilianmds@sc.usp.br (W.d.S.); siqueira@sc.usp.br (A.A.G.S.); 3 I3N & Physics Department, Universidade de Aveiro, Campus Universitário de Santiago, 3810-193 Aveiro, Portugal

**Keywords:** wearable robots, robotic rehabilitation, optical fiber sensors, polymer optical fiber, wearable sensors

## Abstract

Advances in robotic systems for rehabilitation purposes have led to the development of specialized robot-assisted rehabilitation clinics. In addition, advantageous features of polymer optical fiber (POF) sensors such as light weight, multiplexing capabilities, electromagnetic field immunity and flexibility have resulted in the widespread use of POF sensors in many areas. Considering this background, this paper presents an integrated POF intensity variation-based sensor system for the instrumentation of different devices. We consider different scenarios for physical rehabilitation, resembling a clinic for robot-assisted rehabilitation. Thus, a multiplexing technique for POF intensity variation-based sensors was applied in which an orthosis for flexion/extension movement, a modular exoskeleton for gait assistance and a treadmill were instrumented with POF angle and force sensors, where all the sensors were integrated in the same POF system. In addition, wearable sensors for gait analysis and physiological parameter monitoring were also proposed and applied in gait exercises. The results show the feasibility of the sensors and methods proposed, where, after the characterization of each sensor, the system was implemented with three volunteers: one for the orthosis on the flexion/extension movements, one for the exoskeleton for gait assistance and the other for the free gait analysis using the proposed wearable POF sensors. To the authors’ best knowledge, this is the first time that optical fiber sensors have been used as a multiplexed and integrated solution for the simultaneous assessment of different robotic devices and rehabilitation protocols, where such an approach results in a compact, fully integrated and low-cost system, which can be readily employed in any clinical environment.

## 1. Introduction

The advances in rehabilitation therapies have been driven not only by the disruptive technologies with advances in the medicine and robotics fields, but also by the increase in the life expectancy of the general population [1]. Such aging of the population can be linked to some clinical conditions such as stroke, spinal cord injury, Parkinson’s disease and weakness of the skeletal muscles [2]. In addition, other clinical conditions such as cerebral palsy also place demands on new assistive and rehabilitation technologies for the general population (infant, adult and elderly) [3]. In this context, there is a continuous increase in the rehabilitation robots market, which could reach a market size of $6.4 billion by 2025 [4], and in correlated areas such as service robots and smart homes [5].

The advantages of robot-assisted rehabilitation include higher repeatability in the rehabilitation exercises, the possibility of treatment customization and quantitative feedback on the patient’s recovery [1]. Aiming at these advantages, robotic therapy has been applied for lower and upper limb rehabilitation for different pathologies [2] and using different devices [3]. The robots used in the therapy enable the treatment’s customization due to advanced controllers for human–robot interaction, where there is a dynamic interaction between the exoskeleton and the user in order to perform passive, active-assisted and active-resisted movements [4]. These novel technologies and therapy approaches have enabled the evolution of rehabilitation clinics, where there are specialized clinics in robotic rehabilitation [5]. Thus, this evolution in robot-assisted therapy happens due to innovations not only in robot design but also in their control.

In order to achieve a precise and robust control, the robotic devices rely on the kinetic and kinematic feedback from the sensor systems. Conventionally, potentiometers and encoders are employed for the position measurement of wearable robots [6]. However, these sensors need to be carefully attached to the robot’s joints, since they are sensitive to misalignments, which can result in inaccuracies in the sensors’ responses [6]. In addition, the additional structures to position potentiometers and encoders can also result in a bulky or less compact system. As an alternative, regarding the drawbacks of encoders and potentiometers, inertial measurement units (IMUs) are also used for the estimation of a robot’s kinematic parameters. However, their necessity for frequent calibration is a drawback in many applications [7]. It is also worth mentioning that all the aforementioned sensing technologies have sensitivity to electromagnetic fields, which is especially undesirable in robotic applications, where there is a constant activation of electric actuators [8]. The sensitivity to electromagnetic fields is also a drawback for the sensors commonly used in the human–robot interaction force assessment (strain gauges, capacitive sensors and piezoelectric sensors) [6]. Besides the electromagnetic sensitivity, these sensors generally need signal amplification stages and careful attachment to the structure [6].

As the clinical outcomes indicators for robot-assisted rehabilitation have shown impressive results when compared with conventional rehabilitation [2], the cost of the technology still presents challenges for its widespread use. Thus, efforts towards reducing the cost of the wearable devices for rehabilitation is an important concern, which has already been addressed due to advances in additive layer manufacturing (ALM) techniques. Such techniques not only lead to the cost reduction of the robotic device but also to an increase in the customizability of the wearable robot [9]. It is also worth noting that ALM techniques are also an important technology for the development of soft robotics, which are a new generation of compliant robots that can be designed for each user in the so-called human-in-the-loop design [10]. However, most of the conventional sensor technologies are suitable only for rigid structures due to sensitivity to misalignments and the necessity for frequent calibration. Thus, the instrumentation of the novel soft robotics structures possess another challenge for the new generation of soft robots [11].

Optical fiber sensors emerge as an alternative option for instrumentation in different areas, such as structural health monitoring [12], industrial process [13] and medicine [14]. The widespread use of optical fiber sensors occurs due to some advantageous features such as compactness, multiplexing capabilities, electromagnetic field immunity, chemical stability and intrinsic safe operation [15]. If polymer optical fibers (POFs) are analyzed, there are additional advantages related to their material features. These advantages include higher flexibility, higher strain limits, a lower Young’s modulus (leads to higher sensor sensitivity to mechanical loadings) and higher fracture toughness [16], which make these fibers suitable for the instrumentation of soft actuator structures that require a flexible sensing solution with electromagnetic field immunity.

There are many optical fiber sensor technologies, which include sensors based on nonlinear effects [17], fiber Bragg gratings (FBGs) [18], interferometers [19] and intensity variation-based sensors [20]. Most of the aforementioned sensors need equipment such as an optical spectrum analyzer in order to acquire the sensor response. Besides the low portability, such devices have high costs, which will contribute to the increase in the robotic system costs. Compared with FBGs, the proposed sensor system has similar multiplexing capabilities with the possibility of obtaining similar spatial resolutions but with a cost some orders of magnitude lower. It is also worth mentioning that the proposed approach does not need bulky and high-cost equipment for sensor interrogation, which, generally, also presents low acquisition frequency, limiting applications in dynamic movement assessment.

In order to obtain a low-cost solution for exoskeleton instrumentation using POFs, the intensity variation-based principle was used for the development of sensor systems for wearable robots, where the possibility of measuring joint angles [21], human–robot interaction forces [22], ground reaction forces (GRFs) [23] and microclimate conditions [24] was demonstrated. However, as major drawbacks of the intensity variation-based sensors, there is a lack of multiplexing capabilities of these sensors, where each sensor must have its own light source and photodetector [20]. In addition, these sensors present inaccuracies in the measurement when there is any variation on the light source power [20]. Thus, the sensors previously proposed cannot be integrated as a multiplexed solution. In order to tackle this issue, a novel multiplexing technique for intensity variation-based sensors was proposed in [25]. This technique also has a self-referencing system in order to eliminate the effects of light source power deviations on the sensor responses. In addition, compensation techniques based on material features have already been proposed in order to increase the sensor accuracy [26].

Aiming at these advantages, the contribution of this paper is the development of integrated POF instrumentation solutions for lower limb wearable devices. Since there are many alternatives for the use of lower limb exoskeletons for rehabilitation [27], we considered two scenarios: one with the exoskeleton positioned on a stationary base and the other with the person walking in an POF-instrumented treadmill using the exoskeleton. We also considered a third scenario in which, presumably, the person is in an advanced stage of rehabilitation and freely walks with a POF-based sensor system for the simultaneous assessment of gait events, breath rate and GRFs. These scenarios are simultaneously evaluated in order to emulate a robotic rehabilitation clinic fully integrated with POF sensors. To the authors’ best knowledge, this is the first time that integrated optical fiber solutions have been used for the simultaneous assessment of different robotic devices activated at the same time. Such a novel approach opens up new avenues for integrated instrumentation at a robot-assisted rehabilitation clinic, where a single optical fiber cable can be used in the instrumentation of all robotic devices, which leads to a substantial reduction in the cost and size of the systems.

## 2. Instrumentation System Design and Operation Principle

The proposed sensor system is an integrated solution that covers three different scenarios for rehabilitation exercises. The first one is with the Advanced Lower Limb Orthosis for Rehabilitation (ALLOR) positioned on a chair [28], where the user sits on the chair and positions the robotic device on the knee joint. The orthosis has a closed loop impedance control, where there are different degrees of assistance with (or resistance to) flexion and extension movements of the knee joint. In this first scenario, POF sensor systems for angle measurement and human–robot interaction forces are positioned on the ALLOR to quantify the knee angle and interaction forces during the flexion and extension exercises. The same optical fiber cable is used in the second scenario, which is a modular lower limb exoskeleton for gait assistance [29]. The exoskeleton is positioned on the user, and the gait determination is performed on a treadmill with constant velocity. Besides the angle and human–robot interaction forces, the treadmill is also instrumented with POF sensors in order to measure the GRFs and to identify the stance and swing phases of the gait, which are important parameters for the control of gait assistance devices. The third analyzed scenario is the gait analysis of an individual with a wearable sensor. In this case, the POF sensors are positioned on the user, who is asked if they can walk in their desired velocity in a straight path. The sensor system measures the user’s breath rate with a POF sensor positioned on the chest. In addition, the system is also capable of measuring the GRFs on the gait due to POF-instrumented insoles positioned in the user’s shoes.

**Figure 1:**
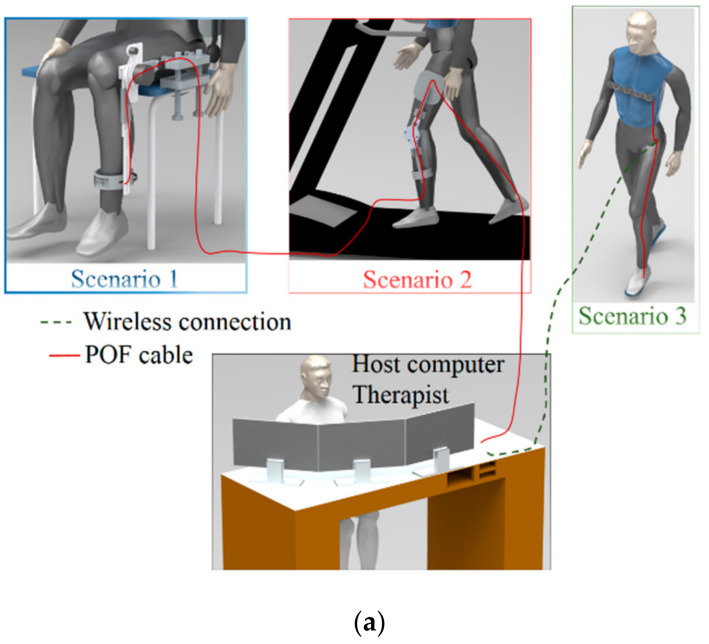

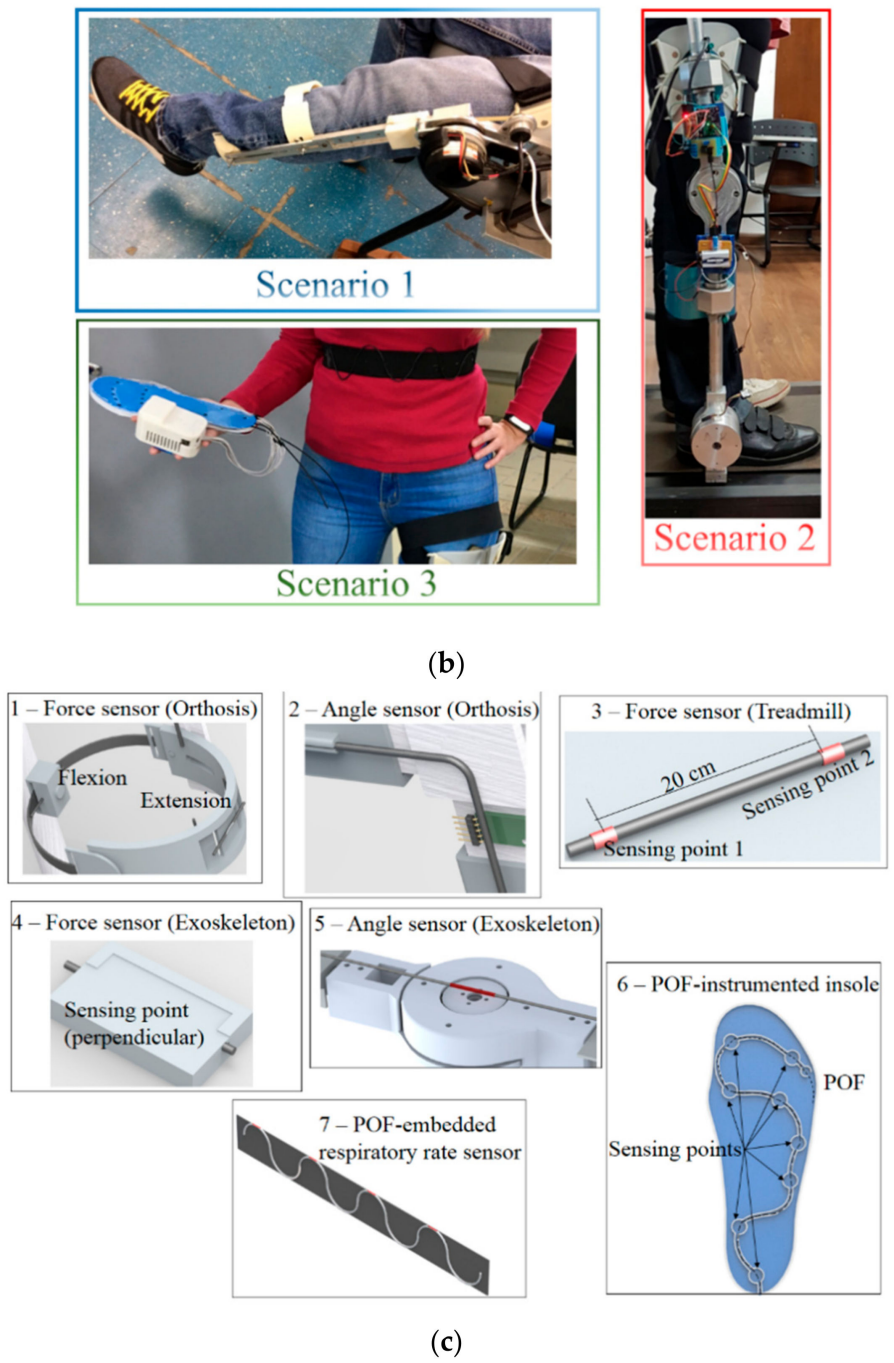
(**a**) Schematic representation of the scenarios proposed, (**b**) a photograph of the devices for each scenario and (**c**) the devices used for the polymer optical fiber (POF)-instrumented robot-assisted rehabilitation clinic.

In summary, the proposed scenarios can be understood as three stages of rehabilitation, where the movement of the knee joint is rehabilitated through flexion/extension cycles (first scenario). Then, in the second stage, the user is able to walk but needs assistance, which is provided by the exoskeleton. In the last scenario, the user is able to walk, but the clinicians need a quantitative analysis of his/her gait in order to compare and analyze the patient clinical evolution. In the context of a rehabilitation clinic, where all the aforementioned rehabilitation approaches will be integrated in the same room, the first (active orthosis integrated on a chair) and the second (exoskeleton for gait assistance on a treadmill) scenarios are instrumented with a single POF cable in order to increase the system integration, compactness and cost-effectiveness. Such a system is linked to a computer to acquire and display the results for the therapist with the additional possibility of sending these results to the cloud through a secured connection for data transmission to another clinic or hospital or remote monitoring in the case of clinics in remote areas. However, for the third scenario (gait analysis with wearable sensors), another POF will be used in order to increase the freedom of movement for the patient. In this case, the data are sent to the host computer through a Bluetooth connection, increasing the portability of the system (see Figure 1a). In Figure 1b, photographs of the setups of each scenario are presented. In this context, POFs offer many advantages when compared with their silica counterparts, where the larger strain limits and fracture toughness of the polymers result in a robust system, which is able to withstand the high strains applied by the dynamic movements. In addition, POFs are more rugged than silica fibers, a feature that enables an easier positioning and attachment of POFs in different structures and/or surfaces. Regarding the tests in each scenario, Scenario 1 has a volunteer positioned on a chair with the orthosis attached to his/her left knee joint, where the user is asked to perform sequential flexion/extension cycles in which the orthosis provides a degree of resistance to the movement. In this case, the POF sensor system acquires the interaction force between the user and the orthosis as well as the curvature angle of the device. In Scenario 2, the user is asked to walk on a treadmill with constant linear velocity, where the attached exoskeleton can provide assistance to the user’s gait. The sensor system for Scenario 2 acquires the angle in the exoskeleton’s knee joint as well as the human–robot interaction forces with the sensor positioned on the shank region. In addition, the treadmill also has POF sensors in order to estimate the GRF of the user’s gait. Finally, Scenario 3 shows the application of the instrumented insole for gait event detection and the smart textile for breath rate monitoring. These sensors measure such parameters while the user walks in a straight line in a sequential 5 m path.

As depicted in Figure 1c, the wearable system for gait analysis comprises three POF sensors: a POF-embedded smart textile for breath rate monitoring and two 3D-printed POF insoles for GRF and gait event assessment. All the POF sensors were fabricated in a polymethyl methacrylate (PMMA) POF with 980 µm core diameter and 10 µm cladding thickness. The light source for the POF sensor system is a light emitting diode (LED) IF-E97 (Industrial Fiber Optics, Tempe, AZ, USA) centered at 660 nm LED, whereas a phototransistor IF-D92 (Industrial Fiber Optics, Tempe, AZ USA) is the photodetector for each sensor. In order to increase the sensor sensitivity, a lateral section is made on the fiber, removing its cladding and part of its core at each detection point [30]. In the POF-embedded smart textile, there are five lateral sections (as indicated in Figure 1b), whereas each POF insole has eight lateral sections, also depicted in Figure 1b. Thus, the POF sensor for the breath rate has four detection points, whereas each insole for GRF monitoring has eight detection points. The analog response of each POF sensor is acquired by the microcontroller FRDM-KL25Z board (Freescale, Austin, TX, USA).

The POF sensor system for the analysis of the first and second scenarios comprises two angle sensors and two interaction force sensors (one for each device). In addition, there is a force sensor on the bottom of the treadmill in order to detect the heel strike and toe off. The integration of the system using a single POF is made through a multiplexing technique for intensity variation-based sensors similar to that depicted in [25]. In this case, each sensor will have its own light source (LEDs similar to the ones used in the wearable sensor system) and each end facet of the POF will be connected to one photodetector. In order to place the light source far from the motors of the robotic devices and treadmill, light couplers IF-562 (Industrial Fiber Optics, Tempe, AZ, USA) are used to couple the light of each LED (we used five LEDs, one for each sensor) to each of the five POF sensors.

Regarding the operation principle of the sensor systems, the intensity variation sensors are based on the variation of the optical power at the fiber as a function of the desired parameter. Therefore, for the instrumented insoles, the plantar pressure/GRFs during the stance phase of the gait leads to curvature and an increase in the stress on the fiber, especially in the lateral sections, which results in the optical power variation detected by the photodetector. The POF-embedded smart textile for breath rate assessment also operates through optical power variation. In this case, the power variation is caused by the expansion and contraction of the chest during the breathing cycles, which is analyzed in the frequency domain through a fast Fourier transform (FFT). This variation results in a stretching of the textile, which leads to the curvature variation of each lateral section of the POF.

**Figure 2:**
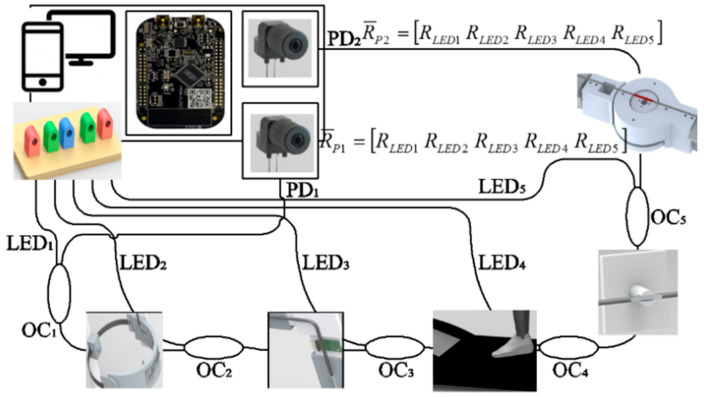
Experimental setup for the POF sensor integration in Scenarios 1 and 2 using the proposed multiplexing technique.

The operation principle of the sensor system for the instrumentation of the first and second scenarios (with different robotic devices) is based on the optical power variation with respect to the angle applied on the POF, for the curvature sensors. If the human–robot interaction force sensors are concerned, the operation principle is similar to that for the other sensors, i.e., optical power variation as a function of the applied force that leads to a curvature and stress increase in the fiber. However, the sensors in this case are embedded in exoskeleton supports in the region where the user applies the forces/torques for the flexion/extension movements. Similarly, the sensors for the instrumented treadmill are positioned on the bottom of the treadmill belt and measure the force through the optical power variation when the foot is in contact with the treadmill belt on the sensitive zone region of the fiber, where the POF used in the treadmill instrumentation has two sensitive zones. For the first and second scenarios, the POF sensor system is integrated in a manner such that the responses of all the sensors are acquired with two photodetectors, one at each end of the fiber cable, as schematically represented in Figure 2.

In order to achieve a multiplexed intensity variation-based sensor system, each sensor is connected to a light source (see Figure 2). Thus, in this case, we have five light sources (two angle sensors, two force sensors and one sensor system for the instrumented treadmill), which are sequentially activated in a predefined sequence, i.e., when LED 1 is activated, the other ones are not active. The sequence of activation is from LED 1 to 5 with an activation frequency of about 30 Hz for each LED, where the microcontroller is responsible for the LED activation and for the signal acquisition when each LED is active. In addition, the sampling rate of the sensor system is 100 Hz with a delay below 10 milliseconds. As depicted in Figure 2, there are two response matrices (R_P1_ and R_P2_) related to the responses of each photodetector, where each column of the matrices is the photodetector signal acquired when each LED is active. Thus, in this case, we have five columns for each matrix, since there are five LEDs, and each LED is connected to one sensor. For this reason, the response of each LED will be related to its corresponding sensor, e.g., LED 1 is related to the response of the POF angle sensor on the active orthosis positioned on a chair. In addition, if the difference between the responses of each photodetector is analyzed, the effects of light source power deviations and environmental variations on the sensor responses are compensated [25].

Even though the photodetector response obtained upon the activation of each LED has a higher contribution of the sensor that is connected to the activated LED, the response can also have the contribution of the other sensors on the POF. In order to obtain the response of each sensor without the influence of the other sensors in the same fiber, it is possible to compensate the response using Equation (1).

(1) $$r_{i}=\frac{R_{i,j}-R_{i-1,j}}{sens_{i}}$$

where *sens_i_* and *r_i_* are the sensitivity and the response of sensor *i*, respectively. Furthermore, *i* is the sensor, i.e., *i = 1, 2, 3, 4* and *5*. In addition, *R_i,j_* is the photodetector response for the photodetector *j* (*j = 1, 2*) when the LED *i* is active. Thus, if the sensitivity of each sensor is known, it is possible to obtain a multiplexed response of the POF sensor system using only one fiber. For this reason, each POF sensor is characterized prior to its application on the multiplexed intensity variation-based system for the proposed POF-instrumented rehabilitation clinic.

## 3. POF Sensor System Characterization

As discussed in Section 2, each sensor needs to be characterized prior to implementation on the multiplexed system in order to obtain the individual response of the sensors without the influence of other sensors (as described in [23,25]). For this reason, each sensor is connected to an LED and a photodetector for the characterization tests. For the POF angle sensors, the POF with the sensitive zone is positioned on a rotary joint with one degree of freedom that provides different controlled angles. The angular range for the characterization of the POF angle sensor positioned on the orthosis for flexion/extension movements is 0° to 90°, due to the physical angle limits of the orthosis. For the angle characterization for the modular exoskeleton, the angular range of 0° to 90° is chosen, since the exoskeleton is used for gait assistance and this range covers the knee angles in the gait cycle.

**Figure 3:**
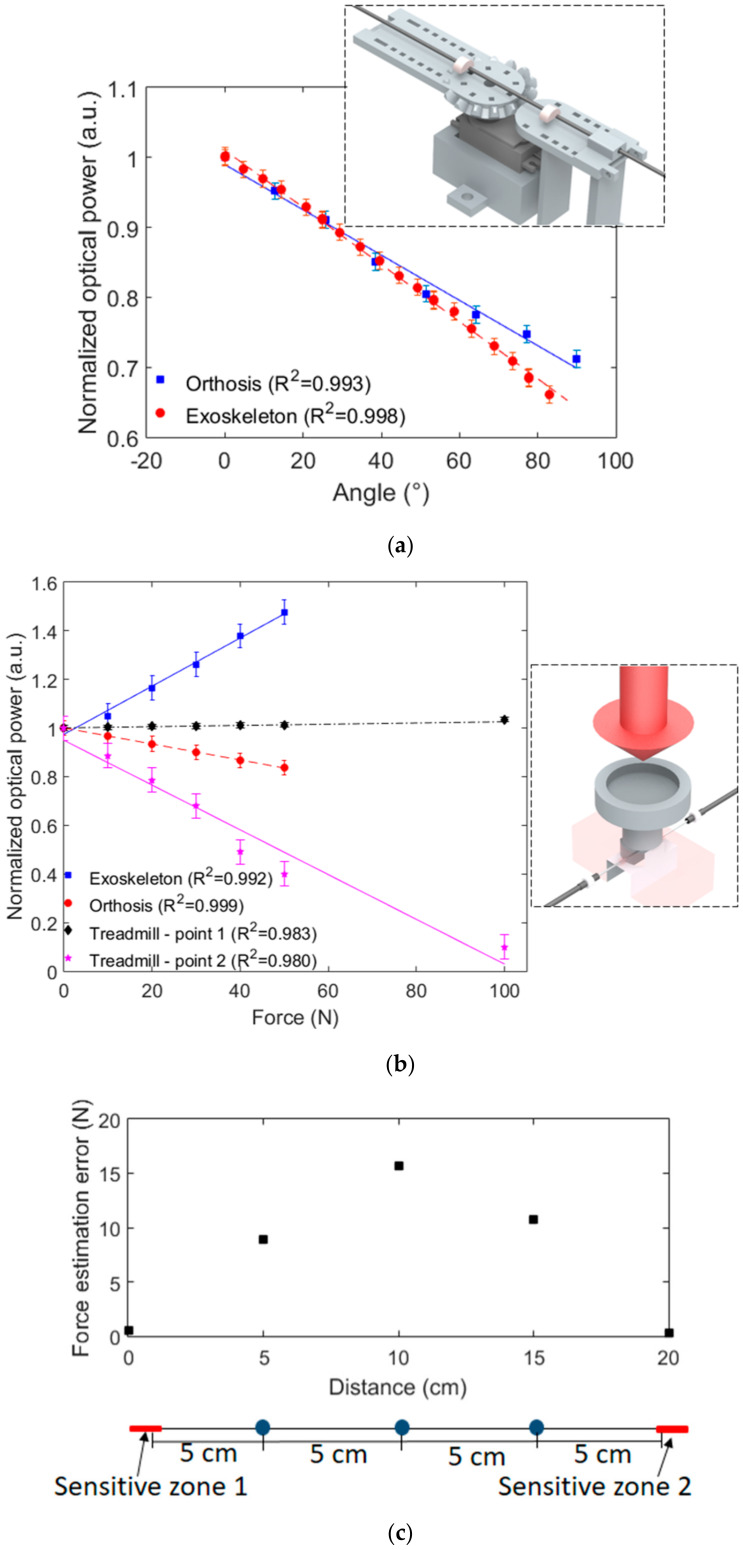
POF sensor characterization as a function of the (**a**) angle and (**b**) force. The figure insets show a schematic representation of the devices used in the characterization. (**c**) Error in the force estimation as a function of the weight placement on the treadmill.

The human–robot interaction force sensors are characterized by means of applying predefined forces from 0 N to 50 N on the sensors through calibrated weights (as shown in the Figure 3 inset). The sensors for the exoskeleton instrumentation are removed from the exoskeleton for the characterization, whereas the force sensors for the orthosis instrumentation are tested within the device’s shank support, since the sensor is already embedded in the structure and cannot be removed. Similarly, the sensor system for the treadmill instrumentation is also characterized by applying constant forces from 0 N to 100 N on the sensitive zone regions. Figure 3 shows the results obtained in the experimental characterization of the sensors used in Scenarios 1 and 2, i.e., two POF angle sensors and two POF interaction force sensors (one for each robotic device) as well as the sensor system for the POF-instrumented treadmill. In this case, Figure 3a shows the responses of the angle sensors, whereas Figure 3b depicts the force sensors. The insets of each figure present the schematic representation of the experimental setup used in the characterization of the sensor systems. In addition, the optical power is normalized as a function of the initial optical power in all cases, and the result is shown with respect to the applied angle or force. Furthermore, an analysis of the force estimation error as a function of the weight placement is shown in Figure 3c, where such an experiment is used to estimate the errors due to deviations in the foot placement on the treadmill. The standard deviations in the sensor characterization in three sequential cycles are also presented in Figure 3. In Figure 3a can be seen a low standard deviation of the sensors when compared with their reference measurements, i.e., the encoder of the exoskeleton and potentiometer on the orthosis knee joint. Regarding the relative error between the encoder, potentiometer and POF curvature sensors, the root mean squared error (RMSE) between the potentiometer of the orthosis and the proposed POF curvature sensor is about 3.3°, whereas the RMSE between the exoskeleton’s encoder and the POF sensor is 1.4°. In both cases, the low RMSE and high R^2^ indicate the suitability of the proposed sensors, where an accurate angle estimation is obtained in the analyzed cases.

The results presented in Figure 3 indicate that each sensor has a linear behavior with a determination coefficient (R^2^) close to or higher than 0.99 in all analyzed cases. Furthermore, it is possible to calculate the slope of each sensor response in order to obtain the sensitivity of each sensor. It is also worth noting that the sensors in Figure 3b have different polarities, i.e., some of them have an optical power increase with the force application and the others, a decrease in the optical power. Such behavior is related to the orientation of the lateral section in each sensor with respect to the point of force application, where different orientations lead to variation not only in the sensor polarity but also in the sensor sensitivity. In addition, the force sensors showed high repeatability in the performed characterization tests, where the standard deviations were as low as 0.05 u.a., which indicates the feasibility of the sensors in repetitive tests. The RMSE of each sensor was also evaluated. Considering the force estimation of each sensor, the lowest RMSE was found in the force sensor for orthosis instrumentation (RMSE of 0.62 N), indicating a high accuracy of such sensors. The POF force sensor for the exoskeleton also showed a low RMSE (1.58 N), whereas the highest ones were found in the treadmill sensors (Points 1 and 2). The analysis of the force sensors in the treadmill shows RMSEs of 2.84 N and 5.42 N for Points 1 and 2, respectively. Such errors can be attributed to minor differences in the weight placement along the optical fiber, which leads to errors in the force estimation. In order to evaluate the error as a function of weight positioning on the treadmill, Figure 3c shows the estimation error when a 100 N force is applied on each point indicated in the figure inset. The results indicate that the error increases as the distance of the weight placement increases. Thus, the highest error is found at the distance of 10 cm, which is the farthest from the sensitive zones. In this case, the errors were as high as 15%; however, it is worth noting that the treadmill has markers for optimal foot placement in the tests and foot positioning at a 10 cm distance from the sensor is unlikely to happen, since this position represents the center of the treadmill and is not normally reached during gait. Moreover, in the treadmill tests, the user has the exoskeleton attached, whose movements occur only in the sagittal plane, which results in an additional constraint for lateral deviations in foot placement on the treadmill.

**Figure 4:**
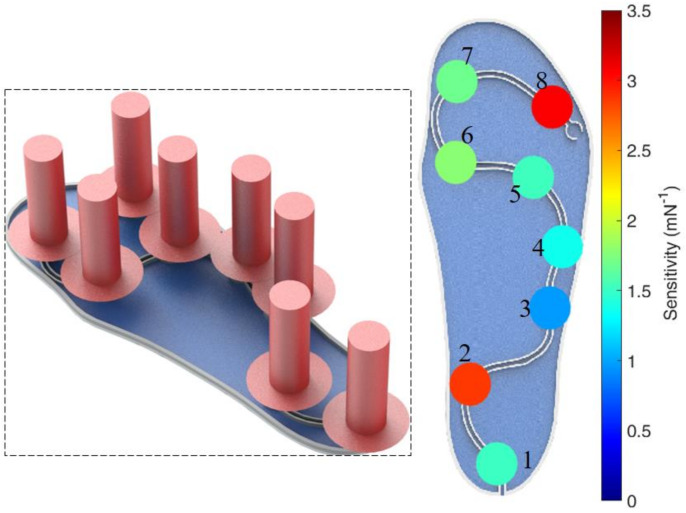
Sensitivity distribution of the POF-instrumented insole. Figure inset shows a schematic representation of the forces positioning in the characterization.

Regarding the sensors for the third scenario, i.e., the POF-instrumented insole and POF-embedded smart textile, the characterization procedure is also applied. In the POF-instrumented insole, the forces (from 0 N to 100 N) are applied at each of the eight sensitive zones produced in the fiber. The applied forces are close to the maximum force at each region as measured in previous studies [23]. In this case, it is important that all the measurement points have similar sensitivities in order to provide similar contributions of each sensor to the GRF curve [31]. Figure 4 shows the sensitivity of each point in the insole characterization; the sensitivities are represented as a colormap in order to provide a better visualization of the sensitivity distribution in the insole. It is worth mentioning that all sensing points presented a linear response in the force characterization, where the determination coefficient is close to or higher than 0.99 in all cases. The differences in the sensors’ sensitivities are due to their positioning on the insole, where some sensors have lower curvature radii and, in some cases, the lateral section in the fiber needs to have a lower length due to the physical restrictions in the insole. For example, the region of Sensor 3 has a lower curvature radius, leading to the lower sensitivity of these sensors. In addition, some minor deviations in the lateral section depth and surface roughness can lead to variations in the sensor sensitivity as discussed in [30].

Despite the high sensitivity of Sensing Points 2 and 8, the sensing points of the instrumented insole presented similar sensitivities between 1.35 mN^−1^ and 1.70 mN^−1^. Since the whole system is connected to the same photodetector, the sensing points need to have similar sensitivities in order to obtain a response in which the GRF curve has a uniform distribution from all the sensing points.

**Figure 5:**
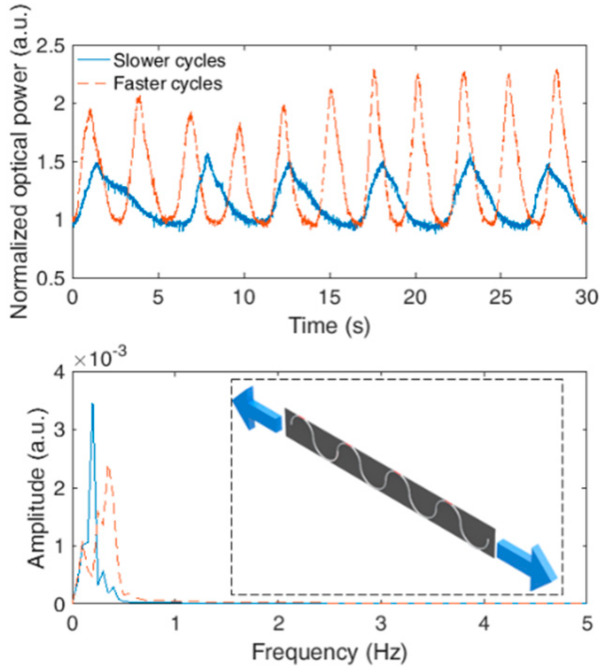
Characterization of the POF-embedded smart textile for respiratory rate monitoring. Figure inset shows a schematic representation of the characterization procedure.

The characterization of the POF-embedded smart textile for breath rate monitoring is achieved by performing oscillatory stretching cycles with a controlled frequency/period for 30 s. The characterization tests are performed at two different rates: six cycles and 11 cycles in 30 s. In order to obtain the respiratory rate, an FFT is applied on the signal and the peak frequency is related to the breath cycles per second. In order to obtain only the frequency response related to the breathing cycles, a second order Butterworth filter is applied on the frequency window of 0.05 Hz to 0.5 Hz, which is the region related to the respiratory rate. Figure 5 shows the characterization in the time and frequency domains for both signals; a schematic representation of the experimental setup is shown in the figure inset. As shown in the time-domain signal, there are six cycles in one test and 11 in the other. If the signal in the frequency domain is analyzed, there is a peak at 0.20 Hz for the first test and one at 0.35 Hz for the second test, which are related to six cycles in a 30 s interval for the first and 10.5 cycles for the second test. These results indicate the feasibility of the proposed approach for estimating the respiratory rate, since the breath leads to a similar periodic response on the sensor (as discussed in [32]).

In general, the proposed sensors presented a high accuracy, with relative errors lower than 3% for all the analyzed sensors, i.e., the breathing rate, GRF for insole and treadmill, human–robot interaction force and angle sensors. It is also worth noting that the sensors presented high repeatability with standard deviations as low as 2% for the force and angle characterizations. For the breathing rate analysis, this deviation is higher. However, it is due to inherent variations in the breathing cycles. Nevertheless, only the frequency response is analyzed for the breathing rate estimation. Thus, such amplitude variations in the time domain do not lead to errors in the breathing rate estimation.

## 4. Implementation of the POF-Instrumented Robot-Assisted Rehabilitation Clinic

After the characterization of the various proposed sensors, they are integrated for the measurements in each scenario. As aforementioned and presented in Figure 2, the sensors for orthosis and exoskeleton instrumentation are integrated in the same system. Thus, the responses of those sensors are acquired simultaneously. In addition, following the characterization performed in Section 3, it is possible to obtain the angle and force responses of the sensors. Moreover, the FFT is applied on the respiratory rate signal in order to obtain the number of breaths during the test in a sample time of 25 s. The response of the instrumented insole is used not only to estimate the GRF curve but also to identify the stance and swing phases as well as detect the gait events on the stance phase by analyzing the GRF curve [31].

For the implementation of the proposed instrumentation methodology, three healthy volunteers (two male and one female, mean age of 29.6 ± 5.5) gave their informed consent for the implementation tests. The tests were performed in accordance with the guidelines of the national health council with the protocols approved by Research Ethics Committee through the National Commission in Research Ethics–CONEP-(Certificate of Presentation for Ethical Appreciation-CAAE: 64797816.7.0000.5542).

**Figure 6:**
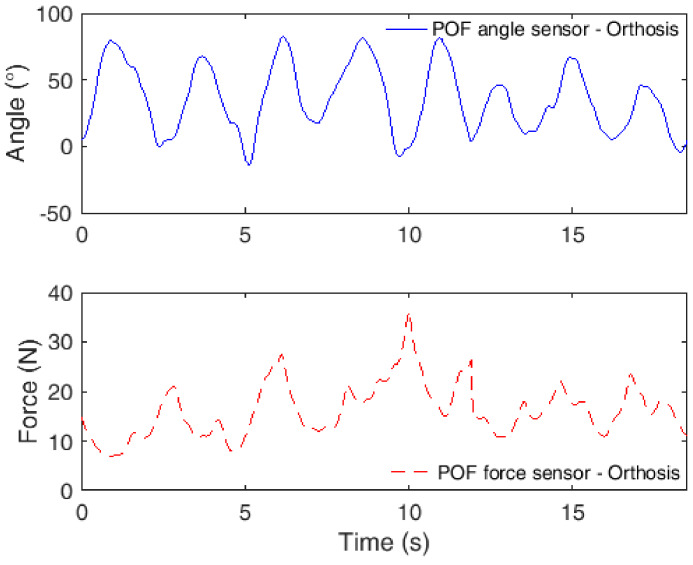
POF sensor responses in robot-assisted rehabilitation using an orthosis for flexion/extension movements.

Each volunteer chooses one scenario, i.e., one volunteer chooses Scenario 1 and the others, Scenarios 2 and 3. One male volunteer is designated for Scenario 1 and the orthosis is positioned on his left leg for sequential flexion/extension cycles. The angle and force responses from the POF sensors are presented in Figure 6 for this test.

In the test, the orthosis only offers resistance to the flexion and extension movements. Thus, it does not move the leg of the user to a predefined angle; it only resists the user’s movement for muscle strengthening. For this reason, there are variations in the maximum angle at each cycle. In addition, it is possible to observe a reduction in the maximum angle in the last peaks, which could be due to the fatigue of the user. However, it is possible to observe angles lower than 0° at 5 s (−5.2°), 8 s (−3.3°) and 19 s (−2.0°). Such errors may be related to misalignments in the orthosis and in the sensors as well; if compared to the force results, the regions at which the angle is lower than 0° are the ones with force increases. Thus, the user forced the orthosis structure to angles below 0°, and, despite the physical limits, the orthosis can show angles lower than 0° due to backlashes in the components and can also lead to misalignments in the POF curvature sensors, leading to the measurement of angles below 0°. Regarding the interaction forces, the results are similar to the ones measured in previous work for the same condition [18], where in the majority of the curves, it is possible to see two peaks: one due to flexion movement and the other for the extension movements. In the last flexion/extension cycles, there is also a decrease in the interaction forces, indicating fatigue of the user.

**Figure 7:**
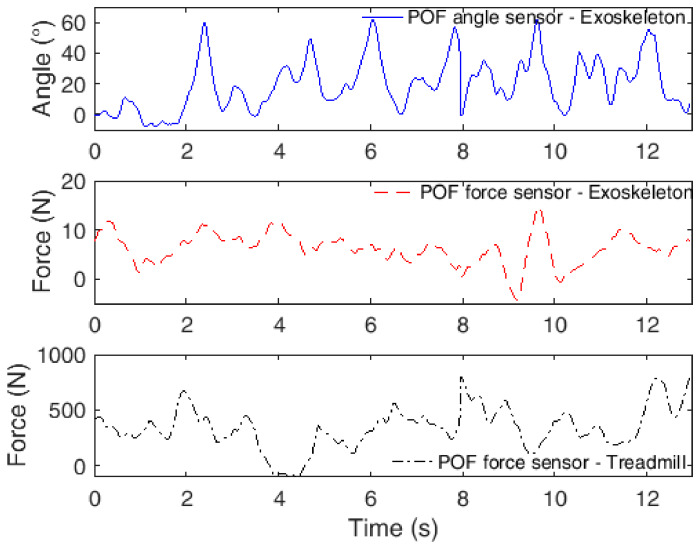
POF sensor responses in robot-assisted rehabilitation using an exoskeleton as a gait assistance device.

Concurrently with the flexion/extension cycles using the orthosis, there is also a robot-assisted gait test using the knee joint of the modular exoskeleton, where both the exoskeleton and treadmill are instrumented with POF sensors. In this case, the other male volunteer is asked to walk on the treadmill at constant velocity for about 5 min. Since each gait cycle has about a 1 s duration, we limited the analysis to 12 s in order to present the cycles in detail. Figure 7 depicts the results obtained with the integrated POF sensor system for angle and force assessment, where the interaction was measured between the human and the exoskeleton as well as that between the human and the treadmill in order to detect heel strike and toe off phases.

As also verified in previous work [18], the human–robot interaction forces are smaller when the robotic device is used for gait assistance. In this case, the forces were lower than 20 N. However, once again, there were more than one peak in each gait cycle, which is related to knee flexion and extension movements during the gait. In addition, the knee angles measured are within the range commonly obtained in gait cycles [33], where the shape of the curve also generally resembles the one obtained for the knee joint during the gait [33]. The force sensors positioned beneath the treadmill belt also presented a local force peak during the heel strike, which occurs at the interface between the ending of a gait cycle and the beginning of another cycle. However, it is also worth noting that in some cycles, the peak value is smaller than in other cycles, which can also be related to the positioning of the foot on the treadmill. If the foot is not in contact with or is far from the region where the sensors were mounted on the treadmill, there will be an attenuated force peak.

**Figure 8:**
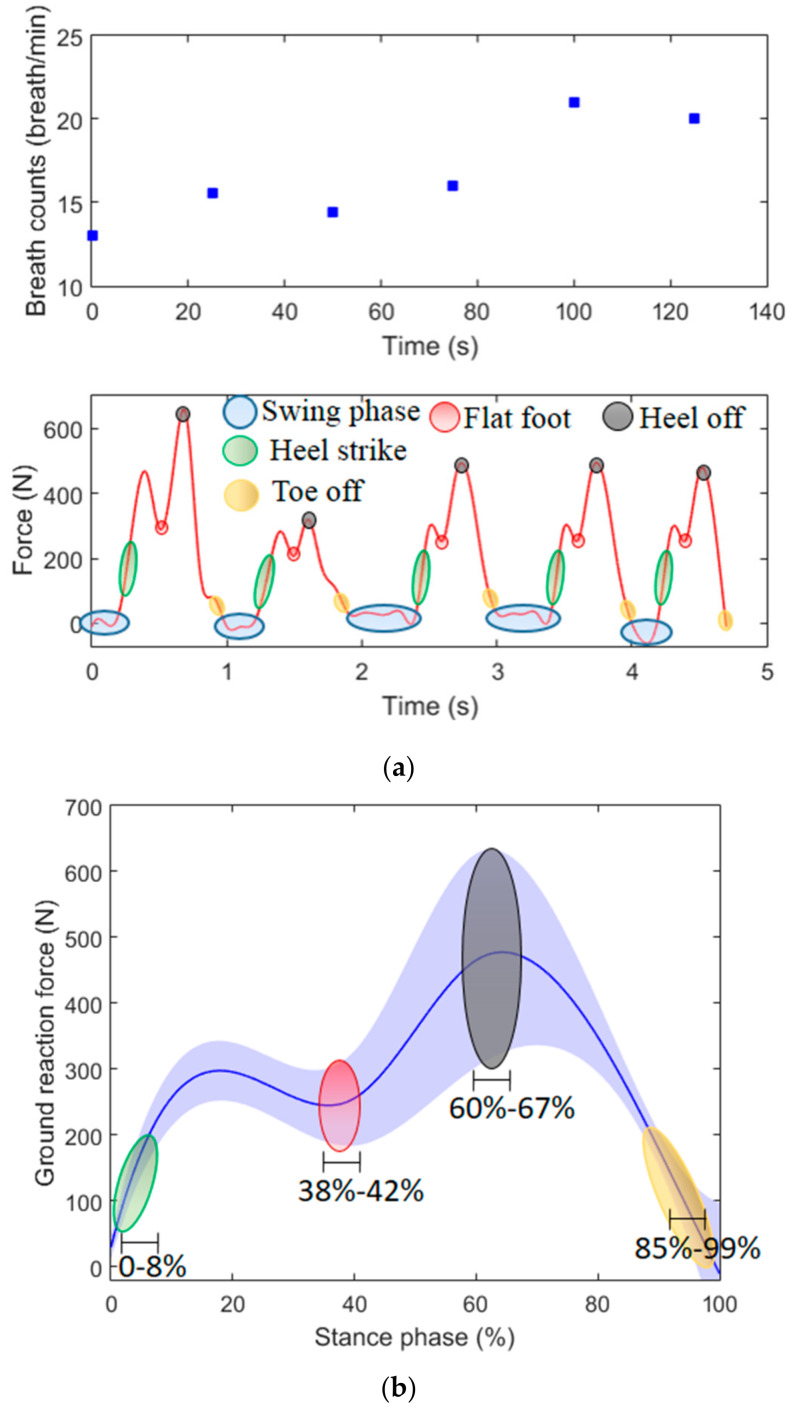
(**a**) Ground reaction force (GRF) and respiratory rate monitoring in gait cycles using POF-based sensor systems. (**b**) Mean (solid line) and standard deviation (shaded line) of the GRF estimated with the proposed insole for the tests in the third scenario. The intervals of the gait events detected are also presented.

In the last analyzed scenario, the female volunteer is asked to walk in a straight path while the POF-instrumented insole and smart textile measure her GRF and respiratory rate, respectively. Once again, the test takes about 5 min in order to also evaluate possible variations in the breath rate due to fatigue. However, we only analyzed five gait cycles with the insole in order to provide more detailed information regarding this parameter and gait event detection. The obtained results in this scenario (free gait in a straight path) are depicted in Figure 8a, where the time span for the analysis of each system is different. For the POF-embedded smart textile, we analyzed the whole test using a sample time of 25 s, whereas for the POF-instrumented insole, we analyzed only five cycles, resulting in a time span of about 5 s. As shown in Figure 8b, the GRF is analyzed for the whole range of the tests, where the solid line represents the mean of the tests, whereas the shaded curve is the standard deviation. The Figure 8b also shows the detection ranges of each gait event.

The respiratory rate results indicate the feasibility of the proposed sensor system for the measurement of such parameters during gait. The volunteer presented a respiratory rate of 13 breaths/minute at the beginning of the test. As the test occurs, the volunteer starts walking in sequential straight paths with different velocities, which can lead to fatigue, resulting in an increase in breath count. It is possible to observe at the end of the test a rate of 20 breaths/minute, which also presented a continuous increase throughout the test, especially after 80 s, when the volunteer was asked to increase her velocity.

The GRF measured with the POF-instrumented insole shows the well-known GRF pattern during the gait, where the peak of the curve is close to 110% of the person’s weight (especially in the three last curves). In addition, it is possible to identify gait events in all performed cycles. In this case, we can identify not only the stance and swing phases but also other gait events in the stance phase such as heel strike, flat foot, heel off and toe off (see Figure 8a). The GRF results in Figure 8b show the possibility of detecting the gait events related to the stance phase, where the heel strike is detected in the slope of the beginning of the test and flat foot showed a range of 38%–42%. Then, the heel off is the second peak in the curve with a range of 60% to 67%. Finally, in the descending curve at the end of the cycle, the toe off is detected. It is worth noting that the large deviations obtained in the sensor can be related to the gait variability in the volunteers. However, it does not inhibit the detection of the gait events, which is the goal of the proposed insole. Such detection is performed by the analysis of the GRF curve variation, instead of the absolute value. The identification of gait events is important, since it can be linked or related to some gait pathologies [31], where the respiratory rate monitoring is also important to indicate the physiological condition of the volunteer during the rehabilitation exercise. Thus, the proposed POF sensor system is a low-cost, portable and compact solution for gait assessment, which can be used not only in the proposed context of a robot-assisted rehabilitation clinic but also in home/remote health monitoring and in the monitoring of daily activities.

## 5. Conclusions

This paper presented the development of an integrated solution based on POFs for the instrumentation of multiple robotic (and non-robotic treadmill) devices for physical rehabilitation as well as wearable sensors for gait analysis. A multiplexing technique for intensity variation-based sensors was used in order to integrate the angle and force sensors for an orthosis (for flexion/extension cycles) and a modular exoskeleton (for gait assistance) in the same POF cable connected to a host computer. In addition, a treadmill with POF force sensors beneath the treadmill belt was used to identify heel strike in the tests using the exoskeleton for gait assistance. For the gait analysis using wearable POF sensors, a POF-instrumented insole for GRF assessment and a POF-embedded smart textile for respiratory rate measurement were employed, where the sensors sent their data using a wireless connection in order to not harm the user’s natural movements. After the sensor characterizations, they were employed in the simultaneous assessment of angle and force on both robotic devices (orthosis and exoskeleton) as well as in force assessment on the treadmill and in gait analysis with respiratory rate monitoring. The results show the feasibility of the proposed integrated POF sensor system in this first proof-of-concept test, which indicates the possibility of future investigations of systems with a higher number of sensors and in an actual rehabilitation clinic. Another future study is the use of the proposed wearable sensors in conjunction with POF angle sensors and even with other sensing solutions (such as IMUs) for joint angles, GRFs and physiological and spatiotemporal parameters of gait.

## References

[1] Kwakkel G., Kollen B.J., Krebs H.I. (2008). Effects of Robot-Assisted Therapy on Upper Limb Recovery After Stroke: A Systematic Review. Neurorehabil. Neural Repair.

[2] Krebs H.I., Hogan N. (2012). Robotic Therapy. Am. J. Phys. Med. Rehabil..

[3] Huo W., Mohammed S., Moreno J.C., Amirat Y. (2016). Lower Limb Wearable Robots for Assistance and Rehabilitation: A State of the Art. IEEE Syst. J..

[4] dos Santos W.M., Caurin G.A.P., Siqueira A.A.G. (2015). Design and control of an active knee orthosis driven by a rotary Series Elastic Actuator. Control Eng. Pract..

[5] Advanced Physical Therapy and Rehabilitation Center, LLC. http://www.aptrehab.com.

[6] Moreno J.C., Bueno L., Pons J.L., Baydal-Bertomeu J.M., Belda-Lois J.M., Prat J.M., Barber R. (2008). Wearable Robot Technologies. Wearable Robots.

[7] El-Gohary M., McNames J. (2012). Shoulder and elbow joint angle tracking with inertial sensors. IEEE Trans. Biomed. Eng..

[8] Leal-Junior A.G., Frizera A., Vargas-Valencia L., Dos Santos W.M., Bo A.P.L., Siqueira A.A.G., Pontes M.J. (2018). Polymer Optical Fiber Sensors in Wearable Devices: Toward Novel Instrumentation Approaches for Gait Assistance Devices. IEEE Sens. J..

[9] Gul J.Z., Sajid M., Rehman M.M., Siddiqui G.U., Shah I., Kim K.-H., Lee J.-W., Choi K.H. (2018). 3D printing for soft robotics – a review. Sci. Technol. Adv. Mater..

[10] Walsh C. (2018). Human-in-the-loop development of soft wearable robots. Nat. Rev. Mater..

[11] Iida F., Laschi C. (2011). Soft robotics: Challenges and perspectives. Procedia Comput. Sci..

[12] Kinet D., Mégret P., Goossen K., Qiu L., Heider D., Caucheteur C. (2014). Fiber Bragg Grating Sensors toward Structural Health Monitoring in Composite Materials: Challenges and Solutions. Sensors.

[13] Diaz C.A.R., Leal A., Marques C., Frizera A., Pontes M.J., Antunes P.F.C., Andre P.S.B., Ribeiro M.R.N. (2019). Optical Fiber Sensing for Sub-Millimeter Liquid Level Monitoring: A Review. IEEE Sens. J..

[14] Mishra V., Singh N., Tiwari U., Kapur P. (2011). Fiber grating sensors in medicine: Current and emerging applications. Sensors Actuators A Phys..

[15] Peters K. (2011). Polymer optical fiber sensors—a review. Smart Mater. Struct..

[16] Leal-Junior A., Theodosiou A., Frizera-Neto A., Pontes M.J., Shafir E., Palchik O., Tal N., Zilberman S., Berkovic G., Antunes P. (2018). Characterization of a new polymer optical fiber with enhanced sensing capabilities using a Bragg grating. Opt. Lett..

[17] Minardo A., Bernini R., Zeni L. (2014). Distributed temperature sensing in polymer optical fiber by BOFDA. IEEE Photonics Technol. Lett..

[18] Leal-Junior A., Theodosiou A., Díaz C., Marques C., Pontes M., Kalli K., Frizera-Neto A. (2018). Fiber Bragg Gratings in CYTOP Fibers Embedded in a 3D-Printed Flexible Support for Assessment of Human–Robot Interaction Forces. Materials (Basel).

[19] Oliveira R., Bilro L., Nogueira R. (2018). Fabry-Pérot cavities based on photopolymerizable resins for sensing applications. Opt. Mater. Express.

[20] Bilro L., Alberto N., Pinto J.L., Nogueira R. (2012). Optical sensors based on plastic fibers. Sensors (Switzerland).

[21] Leal-Junior A.G., Frizera-Neto A., Pontes M.J., Botelho T.R. (2017). Hysteresis compensation technique applied to polymer optical fiber curvature sensor for lower limb exoskeletons. Meas. Sci. Technol..

[22] Leal-Junior A.G., Frizera A., Marques C., Sánchez M.R.A., Botelho T.R., Segatto M.V., Pontes M.J. (2018). Polymer optical fiber strain gauge for human-robot interaction forces assessment on an active knee orthosis. Opt. Fiber Technol..

[23] Leal-Junior A.G., Díaz C.R., Marques C., Pontes M.J., Frizera A. (2019). 3D-printed POF insole: Development and applications of a low-cost, highly customizable device for plantar pressure and ground reaction forces monitoring. Opt. Laser Technol..

[24] Leal-Junior A.G., Díaz C.R., Pontes M.J., Marques C., Frizera A. (2019). Polymer optical fiber-embedded, 3D-printed instrumented support for microclimate and human-robot interaction forces assessment. Opt. Laser Technol..

[25] Leal-Junior A.G., Díaz C.R., Marques C., Pontes M.J., Frizera A. (2019). Multiplexing technique for quasi-distributed sensors arrays in polymer optical fiber intensity variation-based sensors. Opt. Laser Technol..

[26] Leal-Junior A.G., Frizera A., Marques C., Pontes M.J. (2018). Viscoelastic features based compensation technique for polymer optical fiber curvature sensors. Opt. Laser Technol..

[27] Díaz I., Gil J.J., Sánchez E. (2011). Lower-Limb Robotic Rehabilitation: Literature Review and Challenges. J. Robot..

[28] Leal-Junior A.G., Frizera A., Marques C., Pontes M.J., González-Vargas J., Ibáñez J., Contreras-Vidal J.L., van der Kooij H., Pons J.L. (2019). Development of Polymer Optical Fiber Sensors for Lower Limb Exoskeletons Instrumentation. Wearable Robotics: Challenges and Trends.

[29] Dos Santos W.M., Nogueira S.L., De Oliveira G.C., Peña G.G., Siqueira A.A.G. Design and evaluation of a modular lower limb exoskeleton for rehabilitation. Proceedings of the 2017 International Conference on Rehabilitation Robotics (ICORR).

[30] Leal-Junior A.G., Frizera A., José Pontes M. (2018). Sensitive zone parameters and curvature radius evaluation for polymer optical fiber curvature sensors. Opt. Laser Technol..

[31] Leal-Junior A.G., Frizera A., Avellar L.M., Marques C., Pontes M.J. (2018). Polymer Optical Fiber for In-Shoe Monitoring of Ground Reaction Forces During the Gait. IEEE Sens. J..

[32] Leal-Junior A.G., Díaz C.R., Leitão C., Pontes M.J., Marques C., Frizera A. (2019). Polymer optical fiber-based sensor for simultaneous measurement of breath and heart rate under dynamic movements. Opt. Laser Technol..

[33] Kirtley C. (2006). Clinical Gait Analysis: Theory and Practice.

